# Intestinal Obstruction Secondary to Ventral Hernia

**DOI:** 10.4274/balkanmedj.galenos.2019.2018.12.47

**Published:** 2019-05-10

**Authors:** Kamal Kant Sahu, Akil Adrian Sherif, Ajay Mishra, Sunita Vyas

**Affiliations:** 1 Clinic of Internal Medicine, Saint Vincent Hospital, Worcester, United States

To the Editor,

Zheng et al. (1) reported regarding bowel obstruction secondary to obturator hernia, and this study describes our similar experience. We aimed to add to the knowledge bank of Balkan Medical Journal readers regarding abdominal hernias, their spectrum of presentation (subacute intestinal obstruction, strangulation, etc.), and management strategies-medical or surgical (open vs laparoscopic).

An 86-year-old diabetic female was presented to the emergency room with complaints of abdominal pain associated with nausea and vomiting. She reported visiting urgent care several times with similar complaints, and noticed an abdominal bulge over the preceding months. She had an abdominal surgery approximately 30 years ago for acute cholecystitis. Upon presentation, her blood pressure was 116/57 mmHg, pulse rate was 62 per minute, respiratory rate was 18 per minute, temperature was 96.6 F, and oxygen saturation was 100% on room air. Abdominal examination showed a midline scar and bulge in the epigastric region that was soft and nontender on palpation. The skin over the bulge was normal in appearance and normothermic (Figure 1a, b). Laboratory examination showed sodium of 137 mmol/L, potassium of 4.1 mmol/L, chloride of 98 mEq/L, bicarbonate of 22 mEq/L, blood urea nitrogen of 19 mg/dL, creatinine of 1.01 mg/dL, hemoglobin of 12 g/dL, platelet count of 21.000 cells/mm^3^, and a total leucocyte count of 6100 cells/mm^3^. An abdominopelvic computed tomography scan showed dilated small bowel loops up to the ventral hernia sac (Figure 1c-f). Upon diagnosing subacute intestinal obstruction secondary to a ventral hernia, a 16 Fr NG tube was placed through the patient’s right naris, and gastric contents were suctioned out. The patient was medically managed with IV normal saline and antiemetics over the next 48 hours, and gradually tolerated oral fluids with subsequent removal of nasogastric tube. The surgical team performed a laparoscopic mesh repair. Written informed consent was obtained from the patient.

In our case, subacute intestinal obstruction occurred secondary to abdominal adhesions postcholecystectomy. Other common differential etiologies include rectus sheath hematoma, abdominal wall abscess, lipoma, and urachal anomalies (2). Most often an abdominal computed tomography helps to delineate the extent of disease, bowel lumen dimensions, adhesions, congenital anomalies, etc.

Hernias are chronic in nature and typically quiescent until they result in obstruction or sepsis secondary to luminal strangulation. The patients in both cases Zheng et al. (1) and ours-were old-aged with similar symptoms. In our case, the absence of strangulation gave us the window to stabilize the patient first. We followed up with the surgical team who promptly performed a laparoscopic mesh repair. Contrarily, Zheng et al. (1) described a patient with worsened complications secondary to strangulation who required an emergent open surgery.

Abdominal pain is one of the most common reasons for emergency room presentation. Ongoing sepsis, lactic acidosis, and skin discoloration indicates the presence of strangulated hernia and warrants urgent surgical exploration. We firmly believe that both cases together can provide readers with a good understanding of various hernia presentations and treatment strategies (3).

## Figures and Tables

**Figure 1:**
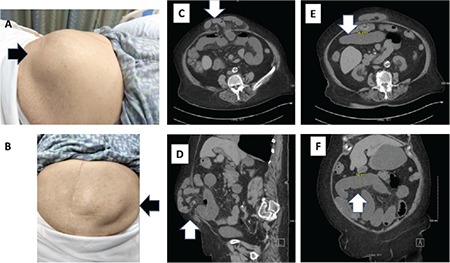
a-f. Abdominal bulge involving the epigastric region: lateral aspect (a) and anterior aspect (b). Dilated bowel loops up to the ventral hernia sac: axial (c) and sagittal (d). Small bowel loop dilation of approximately 3 cm, as indicated: axial (e) and coronal (f).
